# Deciphering the Anti-obesity Benefits of Resveratrol: The “Gut Microbiota-Adipose Tissue” Axis

**DOI:** 10.3389/fendo.2019.00413

**Published:** 2019-06-27

**Authors:** Liyuan Zhou, Xinhua Xiao, Qian Zhang, Jia Zheng, Mingqun Deng

**Affiliations:** Key Laboratory of Endocrinology, Department of Endocrinology, Translational Medicine Center, Peking Union Medical College, Peking Union Medical College Hospital, Chinese Academy of Medical Sciences, Ministry of Health, Beijing, China

**Keywords:** resveratrol, browning of white adipose tissue, brown adipose tissue, gut microbiota, metabolites, obesity, metabolic disorders

## Abstract

Excessive white adipose tissue (WAT) accumulation due to an imbalance between caloric intake and energy expenditure (EE) characterizes obesity. However, brown adipose tissue (BAT) is highly specialized for the dissipation of energy. Recent evidence indicated that the activation of BAT and the induction of WAT browning might be promising approaches to combat obesity by increasing EE and regulating glucose and lipid metabolism. Resveratrol, which is a polyphenolic compound, has been widely acknowledged to have protective effects against obesity and related metabolic disorders. The induction of WAT browning has been considered as one of the crucial factors in the metabolic benefits of resveratrol. Nevertheless, the specific mechanism that is involved is largely unclear. As a prebiotic-like polyphenol, resveratrol is able to modulate the composition of gut microbiota. In addition, in recent years, the impact of gut microbiota on the browning of WAT has received increasing attention and has been initially confirmed to play a role. By considering all these factors, this review explores the potential link between dietary resveratrol and the browning of WAT, which may be modulated by gut microbiota and their metabolites and proposes the “gut microbiota- adipose tissue” axis plays a vital role in the anti-obesity effects of resveratrol. This observation might provide novel insights and targets that could be used for fighting against obesity and associated metabolic disorders.

## Introduction

Obesity has become a worldwide epidemic and has resulted in tremendous economic and social burden. According to the latest data published by the World Health Organization (WHO), the prevalence of global obesity has almost tripled since 1975, and in 2016, more than a third of the adults in the world were overweight or obese (1), which is characterized by abnormal or excessive fat accumulation that might increase the risk of developing a number of disorders, including cardiovascular diseases (2, 3), type 2 diabetes mellitus (T2DM) (4, 5), metabolic syndrome (6, 7), musculoskeletal disorders (8, 9), and some types of cancers (10, 11). An imbalance between caloric intake and energy expenditure is considered as the underlying mechanism of obesity and results in the storage of excess energy in adipose tissue. However, current treatment strategies, such as the modification of the intake and absorption of food and the increase in physical activity, seem to be insufficient to halt the rapid progression of the obesity epidemic (12). Therefore, it is essential to find more effective approaches to fight against obesity and associated cardiometabolic disorders.

White adipose tissue (WAT) is highly specialized for storing lipids and excessively increases its storage capacity during obesity. In contrast, brown adipose tissue (BAT) is an organ that dissipates energy through adaptive thermogenesis (13, 14). Therefore, activating BAT and inducing the conversion of WAT into BAT are considered potential tools to combat obesity by increasing EE and maintaining metabolic homeostasis (15, 16). Although a large number of factors, such as pharmacological and physiological conditions, have been shown to affect the induction of the browning of WAT (17–22), here we will focus on the polyphenolic compound resveratrol. The benefits of resveratrol in obesity and metabolic disorders have been widely acknowledged (23–25). Recent evidence has also suggested that resveratrol supplementation could induce WAT browning and BAT activation (26–28). However, the mechanism by which dietary resveratrol affects adipose tissue remains unclear. In light of the largely hypothetical relationship between resveratrol and gut microbiota (29, 30), and the impact of the gut microbial community on WAT browning (31–33), this review aims to summarize the current literature to explore the association between resveratrol and the browning of WAT through the modulation of the gut microbiota and its metabolites. We propose that resveratrol has an important impact on gut microbiota and the alterations of its metabolites, which in turn influence the regulation of browning and thermogenesis, and this is known as the “gut microbiota-adipose tissue” axis.

## Browning of White Adipose Tissue

Since the last century, the concept of WAT browning has gained increasing attention in academic metabolic research (34, 35). Evidence has shown that mammals have two types of adipose tissue, WAT and BAT, both of which possess unique molecular markers, cell properties, morphology, and function (36).

WAT is distributed throughout the body and mainly comprises two types: subcutaneous adipose tissue (SAT), which is located under the skin, and visceral adipose tissue (VAT), which is accumulated inside the abdominal cavity (37). WAT is highly specialized for the long-term storage of energy in the form of triglycerides (TGs) and fat-soluble substances and provides metabolic fuel in the form of fatty acids, which have great potential to buffer energy intake and consumption. Although WAT is the most plastic organ and can expand its buffering capacity by the hyperplasia or hypertrophy of adipocytes, the energy-buffering capacity would be exceeded by a long-term energy overload (38). In modern life, humans are immersed in the consumption of high-calorie food and live a sedentary lifestyle. Excessive energy intake and reduced energy expenditure leads to excess storage capacity in WAT and further results in lipid overflow from the adipose tissue, which accumulates ectopically in other major metabolic organs and ultimately causes the development of obesity and related metabolic disorders.

BAT is another important type of adipose tissue and is mainly composed of brown adipocytes, which are characterized by larger numbers of mitochondria and smaller fat droplets than WAT (14). The function of BAT has been well studied in rodents (39, 40). However, it was traditionally believed that BAT was only present in newborns and was lost after birth in humans. Recently, multiple studies have demonstrated that BAT can be found in supraclavicular, cervical, perivascular and perirenal areas in adult humans (41–43). BAT specializes in burning nutrients to dissipate energy as heat. BAT has crucial functions in adaptive thermogenesis, which is a significant component of whole-body EE, and is related to cold exposure and food intake (44). Uncoupling protein 1 (UCP1) is uniquely expressed in BAT and is responsible for inducing the adaptive thermogenesis through the uncoupling of mitochondrial oxidation of substrates from ATP production. Thus, the expression of UCP1 is the primary feature of BAT (45). Regulatory factors other than UCP1, including norepinephrine, members of the peroxisome proliferator-activated receptor family (PPAR) (46, 47), PPARγ coactivator 1 alpha (PGC1α) (45), and PRD1-BF-1-RIZ1 homologous domain protein containing protein 16 (PRDM16) (48), also play vital roles in thermogenesis and the development and differentiation of BAT. In light of the important role of EE, BAT has received considerable attention in the treatment of obesity.

Recently, a third type of adipocytes in WAT was identified, known as the beige or brite adipocytes, which has a similar phenotype to brown adipocytes; it has thermogenic features, is rich in mitochondria and expresses high levels of UCP1 and other factors that contribute to thermogenesis (e.g., PPAR, PGC1α, and PRDM16) (49, 50). The presence of brown-like adipocytes in WAT is known as WAT browning (35). In addition to increased EE, BAT, and WAT browning might exert beneficial effects on whole-body metabolism via other pathways, including increasing glucose and lipid clearance and increasing insulin sensitivity by mitigating glucolipotoxicity (51). A current animal study indicated that hyperoxia could improve glucose and lipid metabolism through browning of WAT (52). Some other evidence also showed the association between WAT browning and the improvement in glucose homeostasis, insulin sensitivity as well as lipid mobilization (17, 53, 54). Therefore, the activation of BAT and the induction of WAT browning are promising strategies for fighting against obesity and related metabolic disorders. Other than several pharmacological agents and physiological conditions, such as norepinephrine (55, 56), β_3_-adrenergic receptor agonist (57), exercise (21, 58), and cold exposure (59, 60), that have been shown to activate BAT and induce WAT browning, evidence has indicated that bioactive food ingredients are also key BAT activators and WAT browning inducers that have protective effects against obesity, T2DM and metabolic syndrome (61, 62).

## Dietary Resveratrol, Obesity, and WAT Browning

### Resveratrol and Its Role in Obesity and Related Metabolic Disorders

In recent years, bioactive dietary compounds have received increasing attention in various fields (63, 64). Resveratrol (trans-3,5,4′-trihydroxystilbene) is a natural polyphenol found mainly in plants and plant-derived products, such as *Polygonum cuspidatum*, fruits (including grapes and berries), peanuts and red wine (65, 66). It is estimated that nearly 75% of resveratrol is absorbed after its oral consumption in humans. However, most of it is metabolized in the intestine and liver and the major metabolites are glucuronides and sulfates of resveratrol. Thus, the free form of resveratrol is found at very low levels in serum, thereby decreasing its bioavailability (30). Clinical trials and animal experiments have shown that resveratrol has numbers of health benefits, including the prevention of cancer (67), aging (68), obesity (69), diabetes mellitus (70), and cardiovascular disease (71). Anti-inflammatory and antioxidant activity, improvement in mitochondrial function, and the inhibition of apoptosis, are all considered potential effects of resveratrol (72). This review focuses on the studies that have investigated the potential protective effects of resveratrol against obesity and obesity-related metabolic disorders in animal models and human beings.

#### Evidence From Experimental Animal Models

Evidence from rodents has demonstrated that resveratrol exerts beneficial effects on obesity and related metabolic disturbances (summarized in Table 1). To the best of our knowledge, the anti-obesity effects of resveratrol were first explored in mice in 2006. The research group (73) fed C57BL/6 mice a standard or high-fat diet with or without resveratrol. It was found that body weight and the number of deposits of inguinal, epididymal, and retroperitoneal WAT were significantly reduced in mice that were fed a high-fat diet with resveratrol (200 or 400 mg/kg/day) for 15 weeks. At approximately the same time, another publication (74) demonstrated that resveratrol intake (0.4% in the diet) for 6 months could dramatically improve survival and insulin sensitivity in high-fat diet fed mice, while still being independent of a significant reduction in body weight. Subsequently, numerous beneficial effects of resveratrol, including the reduction of body weight and WAT content and the regulation of lipid metabolism, were also revealed and confirmed in mice and rats fed a high-fat diet (76, 78, 86). However, some experiments showed that body weight was not decreased after resveratrol intake. One study (83) treated C57BL/6J male mice that were fed a high-fat diet for only 3 weeks with oral resveratrol (30 mg/kg/day) and did not observe a significant decrease in body weight or fat mass. The short intervention time might be the main explanation for this. In another study (84), supplementation of a high-fat diet with 0.2 and 0.4% resveratrol for 15 weeks did not reduce body or fat weight in mice. However, similar doses and treatment duration were both identified to have the positive effects in other studies (73, 75). In addition, resveratrol supplementation appeared to have no effect on reducing body weight gain and fat content in rats fed an obesogenic diet (15 mg/kg/day for 6 weeks), which indicated that this dosage was ineffective under this nutritional condition (93).

**Table 1:** Animal studies related with the anti-obesity benefits of resveratrol.

**Animal model**	**Dose**	**Duration**	**Effects**	**References**
C57BL/6J mice high-fat diet	200 or 400 mg/kg/day in the diet	15 weeks	Body weight and fat mass ↓ Adipocyte size ↓ UCP1 and PGC-1α in BAT ↑	(73)
C57BL/6NIA mice high-fat diet	0.4% in the diet	6 months	Insulin sensitivity ↑ PGC-1α and AMPK ↑	(74)
C57BL/6J mice high-fat diet	0.4% in the diet	10 weeks	Body weight gain and VAT weight ↓ Adipocyte size ↓	(75)
C57BL/6J mice high-fat diet	0.005 or 0.02% in the diet	10 weeks	WAT weight ↓	(76)
ApoE deficient mice atherogenic diet	0.02% in the diet	12 weeks	Body weight and epididymal WAT weight ↓	(77)
Kunming mice high-fat diet	200 mg/kg/day in the diet	12 weeks	Body weight and VAT weight ↓	(78)
FVB/N mice high-fat diet	30 mg/kg/day in the diet	60 days	Body weight and adiposity ↓	(79)
C57BL/6J mice high-fat diet	5, 22.5, or 45 mg/kg/day by oral gavage	12 weeks	Body weight ↓	(80)
C57BL/6C mice high-fat diet	1, 10, or 30 mg/kg/day by oral gavage	10 weeks	Body weight gain and WAT weight ↓	(81)
C57BL/6 mice high-fat diet	100 mg/kg/day by oral gavage	4 weeks	TC, HDL-C, ALT,AST, glucose, insulin, and HOMA-IR ↓	(82)
C57BL/6J mice high-fat diet	30 mg/kg/day by oral gavage	3 weeks	No changes	(83)
C57BL/6J mice high-fat diet	0.2 or 0.4% in the diet	15 weeks	No changes	(84)
Sprague-Dawley rats high-fat high-sucrose diet	30 or 60 mg/kg/day in the diet	6 weeks	WAT size ↓	(85)
Sprague-Dawley rats high-fat high-sucrose diet	30 mg/kg/day in the diet	6 weeks	WAT weight ↓	(86)
Sprague-Dawley rats high-fat high-sucrose diet	30 mg/kg/day in the diet	6 weeks	UCP1, SIRT1, and PGC-1α in BAT ↑	(87)
Obese (fa/fa) and lean (Fa/fa) Zucker rats standard diet	10 mg/kg/day by oral gavage	8 weeks	Abdominal fat ↓	(88)
Obese Zucker fa/fa rats standard diet	15 mg/kg/day by an orogastric catheter	6 weeks	Body weight gain and epididymal WAT weight ↓	(89)
OLETF rats standard diet	0.5% in the diet	4 weeks	Body weight gain ↓	(90)
Wistar rats high-fat high-sucrose diet	30 mg/kg/day in the diet	6 weeks	Internal and total adipose tissue weights ↓	(91)
Wistar rats standard chow diet plus cafeteria diet	200 mg/kg/day in the milk	22 days	Body weight and fat accumulation ↓	(92)
Wistar rats high-fat high-sucrose diet	15 mg/kg/day in the diet	6 weeks	No changes	(93)

*UCP1, uncouple protein1; PGC-1α, PPARγ coactivator 1 alpha; SIRT1, sirtuins-1; AMPK, AMP-activated protein kinase; BAT, brown adipose tissue; WAT, white adipose tissue; VAT, visceral adipose tissue; TC, total cholesterol; HDL-C, high-density lipoprotein cholesterol; ALT, alanine aminotransferase; AST, aspartate aminotransferase; HOMA-IR, homeostasis model assessment of insulin resistance; OLETF, Otsuka Long-Evans Tokushima fatty*.

Other than high-fat diets, resveratrol consumption under several other conditions also showed anti-obesity effects. Effects of resveratrol intake (4 g/kg diet) were detected in FVB/N mice fed a high protein diet, where dramatically decreased body weight and adipose tissue weight were shown (94). The same effects were also confirmed in homozygous apoE-deficient mice fed an atherogenic diet with 0.02% resveratrol for 12 weeks (77), obese Zucker rats fed 15 mg/kg/day for 6 weeks or 10 mg/kg/day for 8 weeks (88) and Otsuka Long-Evans Tokushima fatty (OLETF) rats fed a standard diet with 0.5% resveratrol for 4 weeks (90).

#### Evidence From Clinical Studies in Humans

In comparison with animal models, only limited evidence from humans has shown the anti-obesity effects of resveratrol (summarized in Table 2). A randomized, double-blind, placebo-controlled clinical trial analyzed the effects of resveratrol administration (500 mg 3 times per day) for 90 days in 24 patients with metabolic syndrome (95). The results showed that total weight, body mass index (BMI), fat mass, and waist circumference (WC) were all significantly decreased after the intervention, compared with those of the placebo group. Around the same time, another clinical trial detected the effects of resveratrol supplementation (500 mg/day), along with lifestyle intervention in 50 overweight non-alcoholic fatty liver disease (NAFLD) patients for 12 weeks and found significant improvements in weight, BMI, WC, and hepatic steatosis, compared with those of the placebo control group (96). Subsequently, the long-term effects of polyphenols on metabolic health were analyzed (97). Thirty-eight overweight or obese subjects received resveratrol and epigallocatechin-3-gallate supplements (80 and 282 mg/day, respectively) or a placebo for 12 weeks. The VAT mass tended to be decreased in the intervention group compared with the placebo group (*p* = 0.09). Another study also demonstrated that consumption of an orlistat-resveratrol combination for 6 months could dramatically decrease BMI, WC, fat mass, and triglycerides levels in obese subjects compared with those in placebo group subjects (98).

**Table 2:** Clinical trials exploring the anti-obesity benefits of resveratrol.

**Subjects**	**Dose**	**Duration**	**Effects**	**References**
Patients with metabolic syndrome	1,500 mg/day	90 days	Body weight, BMI, WC, and fat mass ↓ AUC of insulin and insulinogenic index ↓	(95)
NAFLD patients	500 mg/day	12 weeks	Body weight, BMI, and WC ↓ ALT, NF-κb, and cytokeratin-18 ↓	(96)
Overweight and obese subjects	EGCG + resveratrol (282 + 80 mg/day)	12 weeks	Visceral adipose weight ↓	(97)
Obese subjects	Orlistat + resveratrol (120 + 100 mg/day)	6 months	Weight body, BMI, WC, and fat mass ↓ TG and leptin ↓	(98)
Obese men	150 mg/day	30 days	Sleeping and resting metabolic rate ↓ TG, ALT, and inflammation markers ↓	(99)
Obese men	150 mg/day	30 days	Adipocyte size↓ Small adipocytes ↑	(100)
Healthy obese men	1,500 mg/day	4 weeks	No changes	(101)
Overweight or obese men with NAFLD	3,000 mg/day	8 weeks	No changes	(102)
Overweight patients with NAFLD	1,500 mg/day	6 months	Liver lipid content ↓	(103)
Non-obese women with normal glucose tolerance	75 mg/day	12 weeks	No changes	(104)

*NAFLD, non-alcoholic fatty liver disease; BMI, body mass index; WC, waist circumference; AUC, area under the curve; TG, triglyceride; ALT, alanine aminotransferase; HOMA-IR, homeostasis model assessment-insulin resistance; EGCG, epigallocatechin-3-gallate*.

Some other clinical trials did not observe the positive effects of resveratrol administration on body weight or fat mass, but they did observe some other metabolic benefits. Two published studies both analyzed the effects of resveratrol administration (150 mg/day for 4 weeks) in healthy obese subjects (99, 100). Although there were no alterations in body weight and fat mass, they found that resveratrol intake could reduce the sleeping metabolic rate and increase the proportion of small adipocytes. Another study also detected the influence of resveratrol consumption on healthy obese subjects, but they used a higher dosage of 1,500 mg/day for 4 weeks (101). The results showed that there were nearly no significant differences in fat mass or energy expenditure. Thus, this dosage might be too high to exert its beneficial effects on metabolism. In addition, several trials evaluated the effects of resveratrol consumption (3 g/day for 8 weeks or 1.5 g/day for 6 months) in subjects with NAFLD and showed that resveratrol intervention had almost no positive effects on the alleviation of clinical or histological NAFLD, and only a small improvement in liver fat accumulation was shown (102, 103). However, a study in which 29 non-obese, postmenopausal women with normal glucose tolerance were administered resveratrol supplementation (75 mg/day) or placebo for 12 weeks and found no changes in body and fat weight or gene expression in WAT (104). Most of the positive effects of resveratrol were observed in obese animals fed an obesogenic diet. Thus, the results of these studies might be due to the fact that they were conducted in non-obese subjects.

Overall, evidence from both experimental animal models and clinical trials in humans confirmed the benefits of dietary resveratrol intake on obesity and related metabolic disorders. Although studies have shown that several metabolic pathways, such as adipogenesis, lipogenesis, apoptosis, lipolysis, thermogenesis, and fatty acid oxidation, might be effective targets for resveratrol, the specific mechanism that explains its effect is not completely understood (69). In light of the important role of WAT browning in the prevention and treatment of obesity (105) and the dramatic anti-obesity effects of resveratrol, this review mainly discusses the mechanisms involved in BAT thermogenesis and WAT browning.

### Resveratrol and WAT Browning

A number of studies have suggested the significant role of resveratrol consumption in the improvement of BAT thermogenesis and WAT browning and its effects on obesity and metabolic health. In addition to observing significant decreases in body and fat weight after 12 weeks of resveratrol intake (400 mg/kg/day), a research group also observed decreases in adipocyte size in WAT and increases in cold-induced thermogenesis, mitochondrial size and the expression of UCP1 and PGC1α in BAT, which indicated a dramatic increase in BAT thermogenesis in mice (73). Another research group treated mice fed a standard diet with resveratrol (400 mg/kg/day) for 8 weeks and found a significant increase in the expression levels of *UCP1, SIRT1* (sirtuins 1), and *BMP-7* in BAT (106). The induction of WAT browning and a significant increase in expression of genes encoding WAT browning biomarkers in WAT, including UCP1, PGC-1α, and PRDM16, were also identified in mice receiving a high-fat diet with 0.1% resveratrol (26). Similar results were observed in inguinal WAT-derived stromal vascular cells after treatment by resveratrol *in vitro* (107).Similarly, a rat model also demonstrated that resveratrol intake (30 mg/kg/day for 6 weeks) in high-fat diet-fed rats significantly upregulated the expression of *PPAR*β*/*δ, *SIRT1* and *PGC-1*α, and UCP1 protein levels in BAT (87). Another animal study showed that supplementation with resveratrol during pregnancy and lactation could result in increased energy expenditure, insulin sensitivity, enhanced brown adipose function, and WAT browning, in male offspring challenged by a high-fat diet (27). A combination of resveratrol and quercetin has been identified to induce a browning-like remodeling effect in perirenal WAT and to increase the expression of UCP1 protein in interscapular BAT (108).

The molecular mechanism involved in the BAT thermogenesis and WAT browning induced by resveratrol was also explored. In 2006, an animal experiment (73) first showed that the resveratrol intake significantly decreased the PGC-1α acetylation and increased its activity, which was consistent with a dramatic increase in SIRT1, which is an important deacetylase. In addition, the effects of resveratrol on BAT were lost in SIRT1^−/−^ MEFs. This finding was confirmed in 2012 with the observation that the induction of WAT browning by resveratrol was dependent on *SIRT1*, which deacetylated the PPARγ and led to the recruitment of the BAT program coactivator PRDM16 (109). In addition, studies demonstrated that resveratrol induced WAT browning through AMP-activated protein kinase (AMPK) α1 (26, 110). Activation of AMPK by resveratrol stimulated mitochondrial biogenesis through SIRT1. The above study showed that resveratrol can induce SIRT1 to further deacetylate PGC1α, which directly regulates the mitochondrial biogenesis and oxidative phosphorylation. Thus, the AMPK-SIRT1- PGC1α pathway might be the key to the induction of WAT browning by resveratrol, which was confirmed again by the resistance to the effects of resveratrol in AMPK-deficient mice (110). In addition, another potential mechanism that mediated the benefits of resveratrol on beige adipocytes was the direct activation of cAMP, which further activates the AMPK pathway. Increased cAMP concentrations in WAT after the oral administration of resveratrol further verified this finding (111). However, the specific mechanism and the direct effects of the activation of the cAMP pathway by resveratrol on WAT browning are still unclear.

A large body of evidence has indicated that resveratrol consumption could induce BAT activation and WAT browning, possibly through the AMPK-SIRT1- PGC1α pathway or the cAMP signaling pathway, and could also have significant benefits for the control of body weight and the improvement of metabolic disorders. However, similar evidence from studies in humans is lacking, and there is a need for more exploration. In addition, the understanding of the specific mechanism by which dietary resveratrol regulates adipose tissue is limited. In light of the important role of resveratrol in the gut microbiota in terms of weight control (112) and the cross-talk between gut microbiota and adipose tissue (113, 114), we further explored whether modifying the gut microbiota via supplementation with resveratrol might promote the BAT thermogenesis and WAT browning.

## Resveratrol, gut Microbiota, and WAT Browning

### Resveratrol and gut Microbiota

The gut microbiota, which is a community of 1,000 or more species of bacteria, that contains 10 times the number of cells in a human and colonizes the gastrointestinal tract, has recently become one of the most popular topics in biomedical research. It has multiple functions in the body, such as absorbing fats and fat-soluble vitamins, digesting complex carbohydrates and plant polysaccharides into short-chain fatty acids (SCFAs), and participating in bile acid-related metabolism. The commensal microbiota in the intestine is in a balanced symbiosis with the host in a healthy host and plays crucial roles in host health (115, 116). During the last few decades, emerging research has focused on the role of the gut microbiota in metabolic diseases and has shown that gut microbiota dysbiosis is intertwined with various disorders, such as obesity (112), glucose intolerance (117), insulin sensitivity (118), and disturbances in serum lipid profiles (119).

Resveratrol, a polyphenolic compound with prebiotic properties, can be metabolized by gut microbiota to produce metabolites, such as dihydroresveratrol and lunularin (30). Interestingly, numerous studies have shown that resveratrol has the potential to modulate the composition of gut microbiota, and this is related to its anti-obesity effects and its improvement of metabolic effects. Effects of resveratrol on gut microbiota were first detected in an obese mouse model induced by a high-fat diet and the results showed that resveratrol intake (200 mg/kg/day) significantly improved the gut microbial dysbiosis in high-fat diet group and decreased body and fat weight (78). The Bacteroidetes/Firmicutes ratios, which is decreased during the development of obesity, and the abundances of *Lactobacillus* and *Bifidobacterium*, were significantly increased, whereas, the abundance of *Enterococcus faecalis* was decreased after resveratrol intervention. Subsequently, another study also showed that the Bacteroidetes/Firmicutes ratio was significantly increased in resveratrol-treated (450 mg/kg/day for 2 weeks) mice with a high-fat diet (120). In addition, increases in the abundances of *Parabacteroides, Bilophila*, and *Akkermansia* and a decrease in the relative abundance of *Lachnospiraceae* were observed in high-fat diet fed mice with resveratrol. A significant increase in the relative abundances of *Bacteroides, Lactobacillus, Bifidobacterium*, and *Akkermansia* and a decrease in the *Prevotella, Ruminococcaceae, Anaerotruncus, Alistipes, Helicobacter*, and *Peptococcaceae* were also identified in mice with trimethylamine-N-oxide (TMAO)-induced atherosclerosis treated with resveratrol (400 mg/kg/day) (121). Subsequently, several experimental animal studies confirmed that resveratrol might regulate body weight and metabolism via modifying gut microbiota (summarized in Table 3). In one study, the results showed that resveratrol intake (0.4%) influenced the gut microbial composition only in obese mice and not in standard diet-fed mice, which was consistent with the positive effects of resveratrol on body weight and metabolism observed in obese animals fed an obesogenic diet (122). However, the evidence from clinical trials in humans is still limited.

**Table 3:** Experimental animal studies analyzing the effects of resveratrol supplementation on gut microbiota composition.

**Animal model**	**Dose**	**Duration**	**Effects**	**Changes of gut microbiota**	**References**
Kunming mice	200 mg/kg/day	12 weeks	Body and visceral adipose weight ↓Blood glucose and lipid levels ↓	Bacteroidetes/Firmicutes ratios, *Lactobacillus*, and *Bifidobacterium* ↑*Enterococcus faecalis* ↓	(78)
C57Bl/6N mice	450 mg/kg/day	2 weeks	Skeletal insulin sensitivity and basal metabolic rate ↑	Bacteroidetes/Firmicutes ratios, *Parabacteroides, Bilophila*, and *Akkermansia* ↑*Lachnospiraceae* ↓	(120)
C57Bl/6J and ApoE^−/−^mice	400 mg/kg/day	1 or 2 months	TMAO-induced atherosclerosis ↓	*Bacteroides, Lactobacillus, Bifidobacterium*, and *Akkermansia* ↑*Prevotella, Ruminococcaceae, Anaerotruncus, Alistipes, Helicobacter*, and *Peptococcaceae* ↓	(121)
C57BL/6N mice	0.4%	8 weeks	Fat mass ↓ Improves glucose homeostasis	*Bacteroides and Parabacteroides* ↑*Turicibacteraceae, Moryella, Lachnospiraceae, and Akkermansia* ↓	(122)
PGC-1α knockout C57BL/6N mice	4 g/kg diet	16 weeks	Body weight ↓	*Erysipelotrichaceae* and *Allobaculum* ↑	(123)
C57BL/6 mice	50, 75, and 100 mg/kg	3 months	Body weight gain, adipose tissue weight ↓ TG, LDL-C, glucose ↓	*Deferribacteraceae* ↑*Coriobacteriaceae* and *Desulfovibrionaceae* ↓	(124)
C57Bl/6J mice	200 mg/kg/day	8 weeks	Body weight gain and fat deposition ↓	*Lactococcus, Clostridium XI, Oscillibacter*, and *Hydrogenoanaerobacterium* ↓	(125)
Wistar rats	400 mg/kg	8 weeks	Fasting blood glucose levels ↓ HDL-C ↑	*Blautia* and *Dorea* ↑*Bacteroides* and *Desulfovibrionaceae* sp ↓	(126)
Wistar rats	Quercetin+resveratrol (30 + 15 mg/kg/day)	10 weeks	Body weight gain and VAT weight ↓ Serum lipids, IL-6, TNF-α, and MCP-1 ↓	*Bacteroidales_S24-7_group, Christensenellaceae, Akkermansia, Ruminococcaceae_UCG-014*, and *Ruminococcaceae_UCG-005* ↑ Firmicutes, *Desulfovibrionaceae, Acidaminococcaceae, Coriobacteriacea, Bilophila*, and *Lachnoclostridium* ↓	(127)
Wistar rats	Quercetin+resveratrol (30 + 15 mg/kg/day)	6 weeks	Body weight gain ↓ Serum insulin levels ↓	Firmicutes/Bacteroidetes ratio, *Erysipelotrichaceae, Bacillus, Eubacterium cylindroides* ↓	(128)
Sprague-Dawley rats	50 mg/L	2 months	Systolic and diastolic blood pressure ↓	Firmicutes/Proteobacteria ratio ↑	(129)

*TMAO, Trimethylamine-N-Oxid; TG, triglyceride; LDL-C, low-density lipoprotein cholesterol; HDL-C, high-density lipoprotein cholesterol; VAT, visceral adipose tissue; IL-6, interleukin-6; TNF-α, tumor necrosis factor alpha; MCP-1, monocyte chemoattractant protein 1; PGC-1α, PPARγ coactivator 1 alpha*.

Fecal transplantation experiments further confirmed the important role of resveratrol-mediated alterations in gut microbiota for the benefits of resveratrol. After discovering that resveratrol supplementation led to changes in the gut microbiota, a research group further explored the causal relationship between the gut microbiota and metabolism through transplanting fecal slurry from healthy resveratrol-fed donor mice to obese mice (122). The results showed that the fecal recipient mice displayed significant improvement in glucose homeostasis, which was independent of the reduction in body weight. The bacterial sequencing analysis showed that the changes in the microbial communities were similar to those observed in the donors. Thus, it is possible that alterations in the gut microbiota mediate the beneficial metabolic effects of resveratrol. Subsequently, effects of fecal microbiome transplants (FMTs) from resveratrol-fed donors (0.4%), for only 2 weeks on the metabolic parameters in recipient mice, were detected (130). Although no changes were identified in body weight and glucose metabolism in the donor mice, the resveratrol-FMTs were sufficient to improve glucose intolerance in the recipient obese mice, also independently of the changes in body weight. In addition, to determine whether live microbes were necessary for the beneficial effects, they measured the effects of heat-killed (HK) FMTs in obese mice. Interestingly, the results showed that HK slurry was also sufficient for the improvement in glucose homeostasis, which indicated that the metabolites or other components in the resveratrol-FMTs were the key factors regulating the metabolic tissues. Recently, another research group (131) transplanted the microbiota from donors treated with a resveratrol (300 mg/kg/day) diet for 16 weeks to the high-fat diet-fed mice, and found that the recipient mice had decreased body weight and improved insulin resistance. In addition, resveratrol-FMTs could induce WAT browning and BAT activation in high-fat diet-fed mice. Therefore, gut microbiota, especially their metabolites, might be a crucial factor in mediating the protective effects of resveratrol against obesity and metabolic disorders, and the modulation of WAT browning by gut microbiota may play an important role.

### The Effects of gut Microbiota on WAT Browning

Recently, several studies have shown the relationship between gut microbiota and WAT browning. It is reported that germ-free mice show stimulation of BAT lipolysis and inhibition of lipogenesis (132). Therefore, they first proposed that gut microbiota could regulate BAT metabolism. A later study analyzed the role of gut microbiota in the energy homeostasis remodeling by cold exposure, which is a well-known BAT activator (133). First, cold exposure significantly changed the composition of the gut microbiota. Then, transplanting gut microbiota from cold-exposed mice to germ-free mice dramatically increased the expression of UCP1 in BAT, increased the number of UCP1-positive cells in inguinal and perigonadal WAT, and improved insulin sensitivity. Therefore, this study indicated that FMTs from cold-exposed mice increased the energy expenditure and induced the browning of WAT. Similarly, in another animal study (134), the exposure of mice to 12°C for 12 weeks could mitigate diet-induced obesity, and this was related to the increased expression of UCP1 in BAT, which was accompanied by significant increases in the abundance of *Adlercreutzia, Mogibacteriaceae, Ruminococcaceae*, and *Desulfovibrio* and decreases in the abundance of *Bacilli, Erysipelotrichaceae*, and the genus rc4-4. Moreover, they transplanted microbiota from donors kept at 12 or 29°C and found that germ-free mice fed a high-fat diet gained less fat mass and showed significantly increased expression of UCP1 mRNA and protein in BAT when colonized with microbiota from donors kept at 12°C instead of donors kept at 29°C. In addition to cold exposure model, intermittent fasting also significantly stimulated the beiging of WAT and improved obesity and insulin sensitivity, and it also produced a shift in the gut microbial composition. Transplantation of fecal microbiota to microbiota-depleted mice induced the browning of WAT while microbiota-depleted mice were resistant to the intermittent fasting-induced browning (32). In addition, the association between the gut microbial community and thermogenesis was also confirmed in a β-klotho KO mouse model, which is naturally resistant to diet-induced obesity due to the stimulation of energy expenditure and BAT thermogenesis (135). In addition, in mice subject to the depletion of gut microbiota by antibiotic treatment and in germ-free mice, the thermogenic capacity of BAT was impaired, and WAT browning was reduced (136). However, only one human study analyzed the relationship between gut microbiota composition and gene markers of browning in SAT and VAT. The results showed that the relative abundance of Firmicutes was positively associated with markers of WAT browning (PRDM16 and UCP1) in SAT (114). These findings all demonstrate the significant role of gut microbiota in BAT browning.

The effects of dietary gypenosides and glucoraphanin on energy metabolism were associated with increased WAT browning and BAT activity, which were accompanied by changes in gut microbiota (137, 138). Polyphenol-rich postfermented pu-erh tea could significantly improve glucose and lipid metabolism, and induced the browning of WAT in high-fat diet fed mice, which was associated with changes in the abundance of *Akkermansia muciniphila* (139). The evidence regarding the role of crosstalk between gut microbiota and WAT browning in the anti-obesity effects of resveratrol is limited. Recently, a study treated high-fat diet fed mice with 0.4% resveratrol for 4 weeks and found that resveratrol could significantly mitigate fat accumulation, promote WAT browning and modulate gut microbiota dysbiosis in high-fat diet-fed mice (28). They then transplanted the feces from these mice into recipient mice and showed that resveratrol-FMTs induced the browning of WAT, in which the SIRT1 signaling pathway might be a key factor. Therefore, this study demonstrated that resveratrol induced WAT browning through modifying gut microbiota.

In light of the crucial role of gut microbiota in mediating the metabolic benefits of resveratrol and the crosstalk between the gut microbial community and WAT browning, we proposed that the “gut microbiota-adipose tissue” axis might be the key to elucidating the anti-obesity effects of dietary resveratrol. Although the link between gut microbiota and WAT browning is unclear, it has been demonstrated that gut microbial metabolites might be a crucial mediator.

## Metabolites Might be a Crucial Mediator Between gut Microbiota and WAT Browning That Plays a Role in the Metabolic Benefits of Resveratrol

### SCFAs

Numerous studies have shown that microbial metabolites might play an important role in the crosstalk between gut microbiota and other metabolic organs, especially the SCFAs (140–144). The SCFAs, which are monocarboxylic acids containing 2–6 carbons, are generated from the degradation of non-digestible carbohydrates by specific bacteria. The major SCFAs in serum and the caecum included acetate, propionate and butyrate, which have been demonstrated to play important roles in metabolic health (145–147), including the induction of WAT browning and the activation of BAT thermogenesis (148, 149). Evidence has shown that the direct supplementation of acetate and butyrate to high-fat diet-fed mice could significantly increase the expression of UCP1 and PGC1α in WAT and BAT (150). In addition, intermittent fasting changed the composition of the gut microbial community, resulting in an increase in the fermentation products acetate and lactate, which could selectively upregulate the expression of monocarboxylate transporter 1 in beige cells and promote WAT browning (32). These effects disappeared in microbiota-depleted mice. Another study also showed that the browning process of WAT was significantly reduced by antibiotic treatment or in germ-free mice, whereas gavage of the butyrate reversed the deleterious effects (136). Thus, the SCFAs were shown to be crucial factors in mediating the effects of gut microbiota on WAT browning. A study in humans showed that plasma acetate levels were positively related to the relative abundance of Firmicutes and were also associated with PRDM16 mRNA levels in SAT (114). An animal study (131) demonstrated that resveratrol significantly increased the relative abundances of *Bacteroidetes, Blautia, Ruminococcus*, and *Parabacteroides*, which have been reported to be SCFA producers. Transplanting the resveratrol-microbiota to high-fat diet-fed mice induced the development of beige adipocytes in WAT, which was accompanied by improved metabolism. In addition, another animal study (130) quantified SCFAs in the fecal matter of the resveratrol fed mice and did not observe significant changes in the levels of major SCFAs, such as butyrate. However, they found an increased concentration the SCFA-4-hy-droxyphenylacetate in the feces, which has been shown to be involved in improving insulin sensitivity and to play a role in WAT browning (151). Therefore, the microbiota derived SCFAs might be vital factors that mediate the anti-obesity benefits of resveratrol in gut microbiota and WAT browning.

### Other Metabolites

In addition to the SCFAs, other metabolites may play a role. Anthocyanins, which were another type of classical polyphenol, have been confirmed to have significant anti-obesity effects; they cannot be absorbed directly but are catabolized by gut microbiota. Vanillic acid (VA) is one of the major metabolites of anthocyanins produced by microbiota. A recent study has shown that VA could reduce body weight, promote the browning of WAT and activate BAT thermogenesis in high-fat diet fed mice (152). KetoA (10-oxo-12(Z)-octadecenoic acid), which is a metabolite of linoleic acid produced by lactic acid bacteria in the intestine, has been shown to mitigate the obesity-related metabolic disturbances and upregulate the expression of UCP1 in WAT via the activation of transient receptor potential vanilloid 1 (TRPV1) (153). Resveratrol, which is a prebiotic-like polyphenol, can be catabolized by gut microbiota into various metabolites, such as dihydroresveratrol and lunularin. This study has indicated that metabolites or other components in the feces of mice given resveratrol supplementation, were sufficient to improve metabolism and induce WAT browning (130). However, the specific metabolites or other components that are involved are still unclear. The evidence for resveratrol-derived metabolites also being crucial mediators of the “gut microbiota-adipose tissue” axis is lacking, and this topic needs further exploration.

Overall, metabolites are critical mediators between gut microbiota and WAT browning and generate the “gut microbiota-adipose tissue” axis, which plays a critical role in the anti-obesity benefits of resveratrol. However, most of the current evidence is from experimental animal models. In the future, more evidence from studies in humans will be required to verify this finding.

## Conclusion

**Figure 1:**
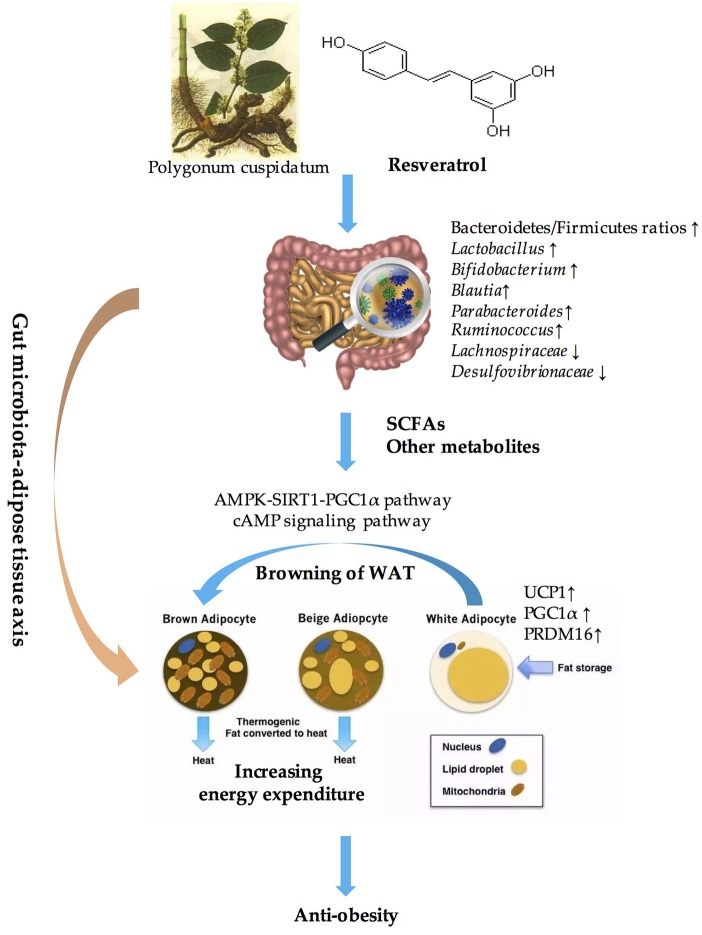
Overview of the role of “Gut microbiota-adipose tissue axis” in the anti-obesity benefits of resveratrol. SCFAs, short-chain fatty acids; AMPK, AMP-activated protein kinase; SIRT1, sirtuin1; UCP1, uncouple protein 1; PGC1α, PPARγ coactivator 1 alpha; PRDM16, PRD1-BF-1-RIZ1 homologous domain protein containing protein 16; WAT, white adipose tissue.

Obesity has become an epidemic worldwide and has presented unprecedented challenges (154). However, the approaches used to fight against obesity and related metabolic disorders seem to be limited. Excessive energy intake and decreased energy expenditure is the main cause of obesity. BAT is highly specialized to dissipate energy as heat, which is an important component of EE. Thus, activating BAT and inducing the browning of WAT to increase EE and maintain metabolic homeostasis might be a powerful means of preventing and treating obesity (34, 35). Resveratrol, which is a polyphenolic compound, has been widely accepted as an anti-obesity agent and a metabolic effector (69, 81, 155, 156). A multitude of evidence has demonstrated that stimulating WAT browning through the AMPK-SIRT1-PGC1α pathway or the cAMP signaling pathway is one of the critical mechanisms that is utilized by resveratrol to combat obesity (157, 158). In addition, resveratrol has been confirmed to have prebiotic properties and was not only metabolized by gut microbiota but also influenced the composition of the gut microbial community (30, 128). The significant role of gut microbiota in inducing WAT browning has also been well established through correlation analysis, fecal transplantation experiments and microbiota-depleted animal models (130, 136, 139). Microbiota-derived metabolites, especially the SCFAs, might be the key to mediating the crosstalk between gut microbiota and WAT browning (130). Therefore, we conclude that resveratrol induces the browning of WAT through modulating the composition of gut microbiota and their metabolites, which play a vital role in anti-obesity effects. In other words, the “gut microbiota-adipose tissue” axis might be the key to elucidating the anti-obesity benefits of resveratrol (summarized in Figure 1).

However, most of the current evidence is from experimental animal models. Because of the enormous differences between humans and animals, especially in terms of the dosages of resveratrol required, more clinical trials are warranted to confirm these findings, as well as the bioactivity and safety of these molecules. In addition, it is necessary to clarify the specific mechanisms involved in the “gut microbiota-adipose tissue” axis that prevent and treat obesity. In this review, we present a new pathway involved in the anti-obesity effects of resveratrol based on the current literature, which provides some potential ideas and targets for the treatment of obesity.

## Author Contributions

LZ: writing-original draft preparation. XX, QZ, JZ, and MD: writing-review and editing. XX: supervision. XX and QZ: funding acquisition.

### Conflict of Interest Statement

The authors declare that the research was conducted in the absence of any commercial or financial relationships that could be construed as a potential conflict of interest.
